# Programme: Texas State Medical Ass’n

**Published:** 1887-04

**Authors:** 


### Society Notes.

### PROGRAMME: TEXAS STATE MEDICAL ASSOCIATION.

The nineteenth annual convention of the Texas State Medical Association will be held in Austin the last week in this month, beginning Tuesday 26th, and continuing four days.

The railroad rate secured for delegates is two cents a mile each way.

On arrival, delegates will be furnished by representatives of the Reception Committee, with cards, giving the name, street address and rates, of the principal hotels and boarding houses. Five hundred of these cards were sent out on 11th. Tuesday morning, delegates will assemble at the Capitol, where they will pay their dues and register; they will then be furnished with badges and certificates of membership.

The exercises will be opened by prayer by Rev. T. B. Lee, Rector of St. David's, Episcopal, Church. An address of welcome will be delivered by the Hon. Jno. W. Robertson, Mayor of Austin, and by Col. T. S. Maxey on behalf of the citizens, and also by a representative of Travis County Medical Association, on behalf of the medical profession of Travis county.

It is impossible to give the order of exercises for each day—as we go to press before Chairmen and Secretaries of Sections can make their reports to the General Committee of Arrangements, inasmuch as papers may be received by them as late as the 15th; but delegates will find, on arrival, completed programmes in pamphlet form, as usual.

The social features of the programme consist of: Tuesday evening 26th, a general reception at Hall of House of Representatives, by the profession of Austin and citizens, assisted by the ladies of the German-American Ladies' Aid Society, with music and refreshments. Wednesday evening 27th, His Excellency Governor Ross will receive delegates and their ladies at the Executive Mansion, from 8 to 12. Thursday evening, President's address at Capitol at 8:30. Friday evening, reception at the University of Texas and at the Institute for the Blind. The managers of the new capitol building will extend invitations to the delegates to visit and inspect the building, going all over the immense structure with them, as guides.

DANIEL'S TEXAS MEDICAL JOURNAL will issue a daily edition during the convention.

